# Adherence as a Predictor of the Development of Class-Specific Resistance Mutations: The Swiss HIV Cohort Study

**DOI:** 10.1371/journal.pone.0077691

**Published:** 2013-10-16

**Authors:** Viktor von Wyl, Thomas Klimkait, Sabine Yerly, Dunja Nicca, Hansjakob Furrer, Matthias Cavassini, Alexandra Calmy, Enos Bernasconi, Jürg Böni, Vincent Aubert, Huldrych F. Günthard, Heiner C. Bucher, Tracy R. Glass, and the Swiss HIV Cohort Study

**Affiliations:** 1 Division of Infectious Diseases and Hospital Epidemiology, University Hospital Zurich, University of Zurich, Zurich, Switzerland; 2 Department of Biomedicine, University of Basel, Basel, Switzerland; 3 Laboratory of Virology, Geneva University Hospital, Geneva, Switzerland; 4 Division of Infectious Diseases, Cantonal Hospital St. Gallen, St. Gallen, Switzerland; 5 Department of Infectious Diseases, Bern University Hospital and University of Bern, Bern, Switzerland; 6 Division of Infectious Diseases, University Hospital Lausanne, Lausanne, Switzerland; 7 Division of Infectious Diseases, University Hospital Geneva, Geneva, Switzerland; 8 Division of Infectious Diseases, Regional Hospital Lugano, Lugano, Switzerland; 9 Institute of Medical Virology, Swiss National Center for Retroviruses, University of Zürich, Zürich, Switzerland; 10 Division of Immunology and Allergy, Centre Hospitalier Universitaire Vaudois, Lausanne, Switzerland; 11 Basel Institute for Clinical Epidemiology and Biostatistics, University Hospital Basel, Basel, Switzerland; 12 Department of Biostatistics, Swiss Tropical and Public Health Institute, Basel, Switzerland; McGill University AIDS Centre, Canada

## Abstract

**Background:**

Non-adherence is one of the strongest predictors of therapeutic failure in HIV-positive patients. Virologic failure with subsequent emergence of resistance reduces future treatment options and long-term clinical success.

**Methods:**

Prospective observational cohort study including patients starting new class of antiretroviral therapy (ART) between 2003 and 2010. Participants were naïve to ART class and completed ≥1 adherence questionnaire prior to resistance testing. Outcomes were development of any IAS-USA, class-specific, or M184V mutations. Associations between adherence and resistance were estimated using logistic regression models stratified by ART class.

**Results:**

Of 314 included individuals, 162 started NNRTI and 152 a PI/r regimen. Adherence was similar between groups with 85% reporting adherence ≥95%. Number of new mutations increased with increasing non-adherence. In NNRTI group, multivariable models indicated a significant linear association in odds of developing IAS-USA (odds ratio (OR) 1.66, 95% confidence interval (CI): 1.04-2.67) or class-specific (OR 1.65, 95% CI: 1.00-2.70) mutations. Levels of drug resistance were considerably lower in PI/r group and adherence was only significantly associated with M184V mutations (OR 8.38, 95% CI: 1.26-55.70). Adherence was significantly associated with HIV RNA in PI/r but not NNRTI regimens.

**Conclusion:**

Therapies containing PI/r appear more forgiving to incomplete adherence compared with NNRTI regimens, which allow higher levels of resistance, even with adherence above 95%. However, in failing PI/r regimens good adherence may prevent accumulation of further resistance mutations and therefore help to preserve future drug options. In contrast, adherence levels have little impact on NNRTI treatments once the first mutations have emerged.

## Introduction

Combined antiretroviral therapy (ART) aims at continuous and lasting suppression of viral replication, which is one of the most important factors influencing long-term prognosis of HIV-infected individuals [1,2]. The importance of adherence to ART has increased as treatment of HIV at present requires life-long therapy once initiated. Non-adherence to therapy has been shown to be one of the strongest predictors of failure of ART [3,4]. Long-term viral suppression requires very high if not perfect adherence, however recent studies have shown that the majority of patients on potent current regimens are able to maintain viral suppression at adherence rates lower than 95% [5–8]. 

Virologic failure is associated with increased risk of emergence of drug resistance [9] and therefore reduces future treatment options and long-term clinical success [10,11]. Studies of the relationship between adherence and resistance in HIV were only conducted a few years ago and indicate that the relationship is more complicated than originally thought, with each drug class having a unique adherence-resistance relationship [12–17]. 

The adherence-resistance relationship in historic monotherapy regimens containing a single unboosted protease inhibitor (PI) or a non-nucleoside reverse transcriptase inhibitor (NNRTI) is thought to be similar. Studies showed that most drug resistance mutations were occurring in individuals with adherence above 90% [10,18–20]. A subsequent mathematical model of PI regimens determined that the maximal resistance occurs at 87% adherence and declines only modestly with perfect adherence [21]. This degree of adherence is low enough to allow for viral failure while high enough to exert selective pressure for resistant virus to emerge. 

Ritonavir boosted PI regimens allow for more potent viral suppression than ritonavir unboosted PIs and this reduces the emergence of resistance mutations. Boosting increases the half-life of the PI and so PI concentrations remain in a suboptimal therapeutic range for a briefer time during periods of non-adherence [18]. Resistance to PIs usually requires multiple mutations and thus exhibit a high genetic barrier; therefore high level resistance requires both ongoing viral replication and sufficient drug exposure to create a selective advantage for drug-resistant virus [22]. 

For NNRTIs, resistance is associated with interruptions in therapy [23] and develops at a lower level of adherence than PI resistance [24]. In addition it has been shown that minority variants harbouring drug resistance mutations can jeopardize therapeutic success of NNRTI but not boosted PI containing regimens [25–27]. Unlike most PI drugs, resistance to the NNRTIs nevirapine and efavirenz requires only a single mutation at the K103N codon and even a single dose of NNRTI monotherapy can result in resistance [28]. In addition, NNRTIs have long half-lives allowing the virus to replicate in the presence of low but detectable plasma levels in the case of consecutive missed doses. Resistance mutations are common in patients with any level of adherence that is insufficient for full viral suppression but almost absent in highly adherent patients. The clinical implications of NNRTI resistance are considerable since NNRTI resistance almost universally confers cross-resistance to first generation NNRTIs and drug resistance mutations persist due to low fitness cost in most cases even after drug discontinuation [29].

The potency of the ART regimen, defined as the likelihood to suppress HIV-1 viremia below the limits of standard assay detection for prolonged periods of time, is the largest single determinant of the development of resistance for all ART classes. The fitness cost of resistance and the genetic barrier to resistance are important but they matter most during active viral replication [13]. Therefore, complete viral suppression and consequently optimized adherence are the undisputed goals of therapy. Adherence patterns of individuals can also have public health implications through the spread of drug-resistant strains of HIV to uninfected or drug-naïve individuals, limiting their future treatment options [30,31]. 

The goal of the study is to quantify the impact of adherence to ART on the development of class-specific resistance mutations in patients starting a new class of ART. 

## Methods

### Ethics statement

Local ethical committees of all seven participating study sites (Kantonale Ethikkommission Bern, Ethikkommission beider Basel (EKBB), Ethikkommission des Kantons St. Gallen, Kantonale Ethikkommission Zürich, Comitato etico cantonale del Ticino, Commission d'éthique de la recherche clinique de la faculté de biologie et de medicine de l'université de Lausanne, Comité d'éthique du départment de médicine des hôpitaux universitaires de Genève) have approved the study and written consent has been obtained from all participants.

### Selection of patients and genotypic drug resistance tests

Patients for this study were selected from the SHCS, which is a nationwide observational study of HIV infected individuals in medical care in Switzerland. In semi-annual visits, laboratory measurements are performed and clinical questionnaires are administered [32]. The genotypic drug resistance data stem from the SHCS drug resistance database, which is a central, anonymized collection of all genotypic drug resistance tests ever performed on SHCS enrolees by one of the four authorized laboratories in Switzerland [16], complemented by a systematic retrospective collection of pre-treatment genotypes [33]. The data are stored in SmartGene’s Integrated Data Network Services tool (Version 3.6.1).

**Figure 1:**
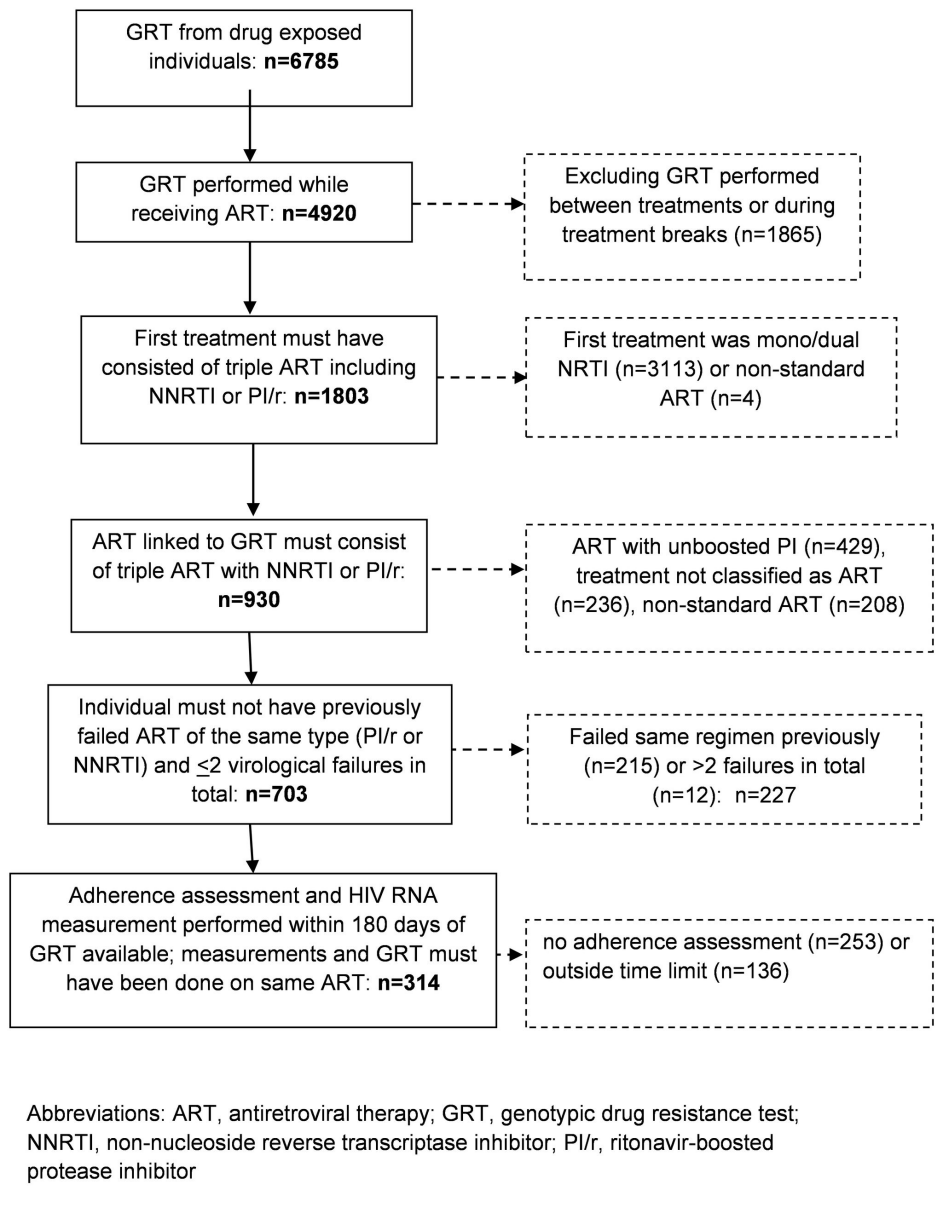
Selection of genotypic drug resistance tests.

The SHCS drug resistance database was screened for genotypic drug resistance tests (GRT) which were performed while patients were receiving ART and to which an adherence assessment could be linked. In particular, GRTs/patients were included if the following conditions were fulfilled (Figure 1): Patients had to be either 1) therapy naïve individuals initiating either a ritonavir-boosted PI (PI/r) or a NNRTI regimen or 2) if having failed a first regimen with an on-treatment HIV RNA >500 copies/mL after 24 weeks of therapy, they must have initiated a second regimen with two fully active drugs (corresponding to a Stanford genotypic sensitivity score < 2) that contained a different third drug class (i.e., NNRTI or PI/r) than the initial regimen. Moreover, GRT included in the analysis had to be performed after a minimum ART exposure duration of 30 days, be linked to a completed adherence questionnaire within 180 days of the genotype, and an HIV RNA measurement must have been obtained on the same treatment regimen. These conservative selection criteria were designed to maximize sample size while only measuring newly emerging drug resistance mutations against antiretroviral therapies that were functional at the time of initiation. We further aimed to exclude salvage therapies, because the origin of mutations and consequently the impact of adherence can often no longer be assessed accurately in this population. Hence, GRTs performed on patients receiving integrase inhibitors or CCR5 entry inhibitors were also excluded from this analysis because in Switzerland these drugs were only used in trials of salvage treatments during the observation period. 

#### Adherence

On twice-yearly study visits, levels of self-reported adherence are assessed via interview with the clinician. Patients are asked about the frequency of missed drug doses over the past four weeks (never, less than once a month, monthly, twice a month, weekly, more than once a week, daily) and whether consecutive doses of ART were missed. Using the first question plus the dosing frequency of the regimen, the percentage adherence was calculated and then categorized into 100%, 95-99%, and <95%. We considered the inclusion of both adherence measures (percentage adherence and consecutive missed doses) together as well as separate in all models. The predictive value of the adherence questions and non-adherence definitions with regard to viral load levels have been demonstrated previously [3].

### Statistical analysis

The primary study outcome was the new emergence of any International AIDS Society – USA drug resistance mutations while receiving antiretroviral therapy [34]. The primary explanatory variable of interest was adherence to antiretroviral therapy over the past 6 months, stratified into the following categories: 100%, 95-99%, and<95% adherence. In order to test the independent association of adherence with emergence of drug resistant mutations, multivariable models were constructed adjusting for the following pre-specified potential confounders: age, gender, active or prior injecting drug use, AIDS-defining events, baseline CD4 cell count, baseline HIV-1 RNA, and the nucleoside reverse transcriptase inhibitor (NRTI) backbone combination (in particular the inclusion of 3TC, a low genetic barrier drug) and number of previous regimens. Univariable and multivariable logistic regression models were performed separately for NNRTI and PI/r. Unless a significant departure from linearity was detected by likelihood ratio tests, levels of adherence were primarily fitted as linear trends across the pre-defined strata.

In addition to the analysis of any new IAS-USA mutations, further sub-analyses considered specific types of mutations, which were any major mutations to the group-specific third drugs (i.e. either PI/r or NNRTI) and the new emergence of M184V mutations. The latter mutation was selected due to its low genetic barrier and the widespread use of M184V selecting drugs in Switzerland. These sub-analyses were restricted to individuals with the appropriate drug exposures, which selected for the mutations of interest. As a secondary outcome, we compared levels of plasma HIV RNA around the time of the adherence assessment across the different strata of medication adherence. 

Because a number of included patients lacked information from prior genotypic drug resistance testing (Table 1) we performed sensitivity analyses by repeating our analyses on the subset of individuals who either received their first ART ever or those who started a new line of ART (including one new drug class) and who had a prior on-treatment genotypic resistance test done.

**Table 1:** Baseline characteristics of population by drug class.

**Variables**	**Category**	**PI/r**	**NNRTI**
N (%)		152 (100.0)	162 (100.0)
Female – n(%)		46 (30.3)	41 (25.3)
Age, median [IQR]		40 [35 to 46]	39 [33 to 46]
Ethnicity – n(%)	White	112 (73.7)	110 (67.9)
	Black	27 (17.8)	39 (24.1)
	Other	13 (8.6)	13 (8.0)
Mode of HIV acquisition – n(%)	Heterosexual	61 (40.1)	86 (53.1)
	Intravenous drug use	24 (15.8)	11 (6.8)
	Homosexual	65 (42.8)	59 (36.4)
	Other	2 (1.3)	6 (3.7)
Previous or current IDU – n(%)		26 (17.1)	12 (7.4)
Year of ART initiation, median [IQR]		2003 [2000 to 2006]	2002 [1999 to 2005]
Baseline CD4, n, median [IQR]		140; 218 [101 to 360]	158; 190 [78 to 307]
Baseline HIV RNA, n, median [IQR]		138; 5.1 [4.8 to 5.7]	150; 5.1 [4.6 to 5.7]
Ever virologically failed ART		2 (1.3)	22 (13.6)
Ever failed 3TC		0 (0.0)	18 (11.1)
Ever failed other NRTI		2 (1.3)	22 (13.6)
Ever failed PI		0 (0.0)	20 (12.3)
Ever failed AZT or d4T		1 (0.7)	22 (13.6)
Number of previous lines of ART- n(%)	0 (First line treatment)	45 (29.6)	57 (35.2)
	1	47 (30.9)	44 (27.2)
	2	25 (16.4)	21 (13.0)
	3	22 (14.5)	19 (11.7)
	4	5 (3.3)	11 (6.8)
	5 or more	8 (5.3)	10 (6.2)
Prior RT- n(%)	Results available	114 (75.0)	98 (60.5)
	With GSS >2.5 at baseline	112 (98.3)	95 (96.9)
Time between adherence assessment and genotypic testing, median number of days, [IQR]		0 [-8.5 to 19]	0 [-11 to 34]
Adherence - n (%)	100%	113 (74.3)	108 (66.7)
	95%-99%	18 (11.8)	28 (17.3)
	90%-94%	15 (9.9)	18 (11.1)
	85%-89%	1 (0.7)	3 (1.9)
	<85%	5 (3.3)	5 (3.1)
HIV RNA at genotypic test, median [IQR]		2.7 [2.3 to 3.8]	3.5 [2.9 to 4.6]
CD4 cell count at genotypic test, n, median [IQR]		124; 351 [238 to 494]	136; 377 [215 to 553]

IQR=interquartile range, IDU=injecting drug use, ART=antiretroviral therapy, NNRTI= non-nucleoside reverse transcriptase inhibitor, PI/r=ritonavir-boosted protease inhibitors, NRTI= nucleoside reverse transcriptase inhibitor, 3TC= lamivudine, AZT=zidovudine, d4T=stavudine, RT=resistance test, GSS=genotypic sensitivity score

All analyses were done using SAS v9.2 (SAS Corporation, Cary, NC, USA) and Stata v12.0 (StataCorp LP, College Station, TX, USA). All p-values are two-sided and the threshold for statistical significance was set at 0.05.

## Results

### Patient Characteristics

Of over 6700 GRT from therapy-exposed individuals, we were able to select 314 test results belonging to 162 individuals receiving NNRTI and 152 receiving PI/r (Figure 1). Patient characteristics are displayed in Table 1. In the NNRTI group, 72% of all individuals received efavirenz (EFV), 27% nevirapine (NVP) and 1% etravirine. The most frequent PI used in the PI/r group was lopinavir (55%), followed by atazanavir (39%). The nucleoside reverse transcriptase inhibitor backbones contained either lamivudine (3TC) or emtricitabine (FTC) in 90% (PI/r) and 84% (NNRTI) of individuals, thymidine analogues in 30% (PI/r) and 46% (NNRTI), tenofovir (TDF) in 53% (PI/r) and 44% (NNRTI), and abacavir (ABC) in 18% (PI/r) and 14% (NNRTI) of all regimens. 

Among the most notable differences between the two groups were the somewhat higher proportion of individuals who have acquired HIV via injecting drug use in the PI/r group and the lower proportion of individuals in the PI/r group who had previously experienced virological failure on antiretroviral therapy. Levels of adherence were similar between the PI/r and NNRTI groups, and approximately 85% of individuals reported adherence levels >95% in both groups. Due to the limited number of cases, all adherence strata <95% were collapsed into one group for further analyses. 

### Genotypic sensitivity score

As shown in table 2, levels of drug resistance were considerably lower in the group of PI/r recipients – for example, resistance to any IAS-USA mutations was detected in 56 (34.6%) of NNRTI recipients versus 14 (9.2%) of PI/r recipients (p<0.001). These findings were confirmed by sensitivity analyses (Table S1).

**Table 2:** Development of new mutation by adherence level and drug class.

**Adherence Level**	**PI/r**	**NNRTI**
**Any IAS-USA mutation**		
100%	10/113 (8.9)	31/108 (28.7)
95-99%	1/18 (5.6)	12/28 (42.9)
<95%	3/21 (14.3)	13/26 (50)
**Any mutation against group-specific drug (PI/r or NNRTI)**		
100%	3/113 (2.7)	27/108 (25)
95-99%	1/18 (5.69)	8/28 (28.6)
<95%	0/21 (0.0)	11/26 (42.3)
**New emergence of M184V/I**		
100%	1/106 (1.0)	13/86 (15.1)
95-99%	0/13 (0.0)	5/24 (20.8)
<95%	3/18 (16.6)	5/22 (22.7)

NNRTI= non-nucleoside reverse transcriptase inhibitor, PI/r=ritonavir-boosted protease inhibitors

Indeed, the Stanford cumulative genotypic sensitivity scores of the drug combination at the time of genotyping (i.e. the number of active drugs) were generally higher in the PI/r group (median [IQR], 3 [3–3]) than in the NNRTI group (2 [1-3], Wilcoxon rank sum p<0.001; data not shown). Results for the sensitivity analysis were identical (data not shown).

Moreover, when comparing cumulative genotypic sensitivity scores upon failure across adherence strata, there was a trend for lower scores (i.e. more resistance) with lower adherence in the PI/r group (reduction of -0.09 [-0.20; 0.01] score points with decreasing adherence, p=0.074, data not shown). No such trend was observed for the NNRTI group. Potentially those results in the PI/r group could have been influenced by previous virological failures, which may have affected genotypic sensitivity scores for NRTI drugs (not PI/r or NNRTI scores, however). Nevertheless, those associations between GSS and adherence in the PI/r were still observed when repeating the analysis on the set of individuals starting their first antiretroviral therapy (-0.17 [-0.30; -0.03], p=0.016; data not shown).

### Associations of emergence of drug resistance with adherence

Descriptive analyses shown in Table 2 indicate that adherence may indeed influence the probability of detecting IAS-USA mutations by genotypic testing. In the group of NNRTI recipients there was a steady increase in the proportion of tests with resistance mutations for all three outcomes - the presence of any mutations, NNRTI mutations, or M184V mutation. This trend persisted even in an analysis of adherence categorized into the five original strata (not shown). Increases in proportions of resistant viruses with lower adherence levels were observable when analysing any IAS-USA mutations or M184V as outcomes, but not with the emergence of PI mutations. However, when performing sensitivity analyses with a more strictly defined population who either had started their first ART or with a prior on-treatment GRT available(Table S1) those associations of adherence levels with emergence of new NNRTI or M184V mutations were no longer apparent, possibly owing to the lower sample size.

**Figure 2:**
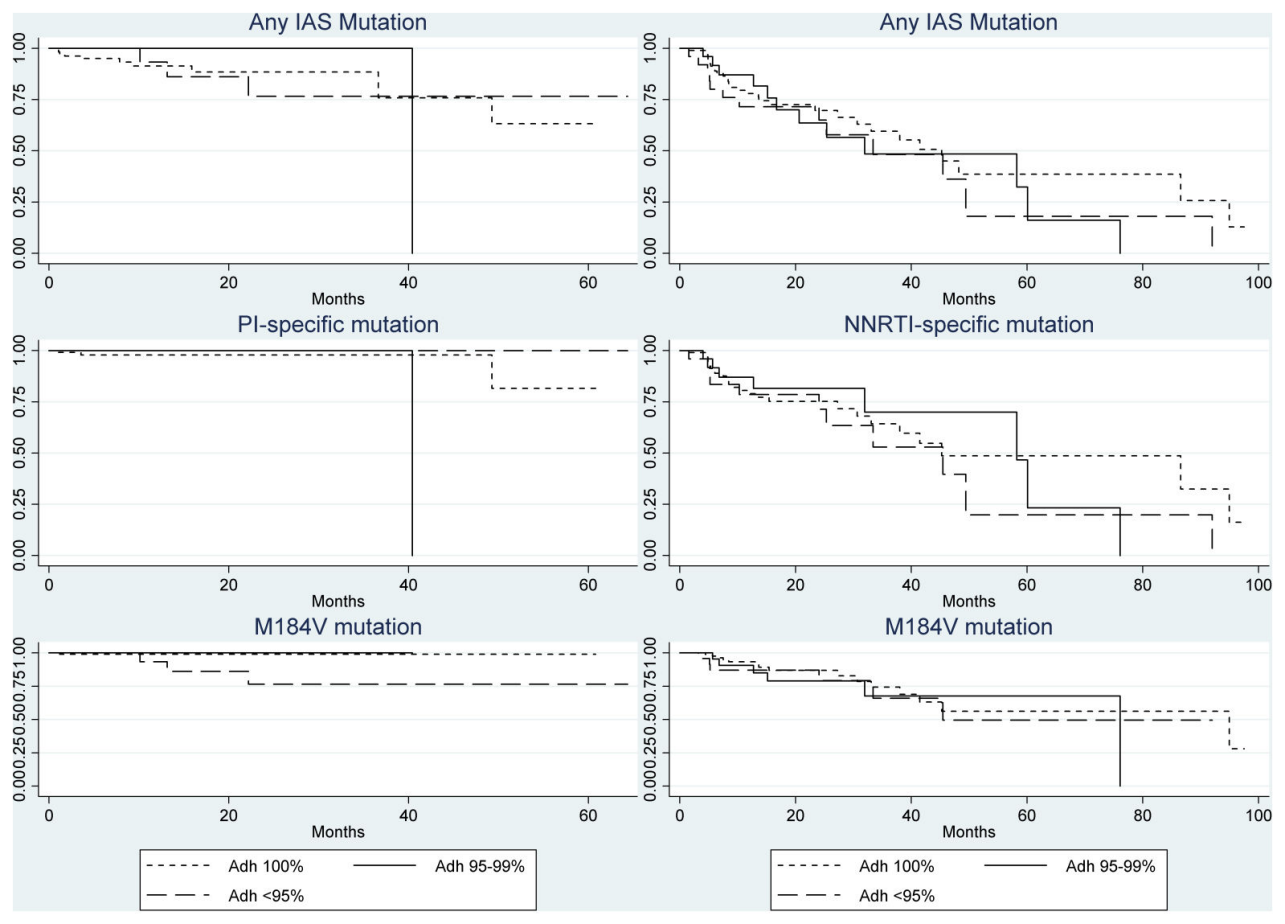
Kaplan-Meier curves for the association of adherence on the development of new resistance mutations. The left column is those on PI/r regimens (N=152) and the right column is those on NNRTI regimens (N=162).

Kaplan-Meier curves for the unadjusted association between adherence and development of mutations were estimated (Figure 2). These observed adherence-resistance relations were further tested for statistical significance in univariable and multivariable logistic regression models. In univariable analyses, the only outcome showing a statistically significant association with adherence in the PI/r group was the emergence of the M184V mutation (n=137 patients on 3TC or FTC backbone, odds ratio [95% confidence interval]: 4.98 [1.41-17.62]) per increase in adherence stratum (Table 3). This estimate should be interpreted with caution, however, because the M184V mutations did not occur in the middle adherence stratum. In multivariable models, adherence was still significantly associated with development of a new M184V mutation (8.38 [1.26-55.70], p=0.03), however the confidence intervals are very wide and reflected the small sample size of this subgroup. 

**Table 3:** Multivariable logistic regression models for the development of mutations in individuals on PI/r regimens.

**Variable**	**Any IAS-USA mutation**		**Any PI/r mutation**		Any M184V/I mutation*	
	**Multivariable OR**	**p-value**	**Multivariable OR**	**p-value**	**Multivariable OR**	**p-value**
Female	0.94 (0.25 - 3.55)	0.92	2.01 (0.19 - 20.69)	0.56	0.45 (0.03 - 6.69)	0.57
Age - per 10 years	0.88 (0.45 - 1.75)	0.72	0.74 (0.19 - 2.88)	0.66	0.55 (0.10 - 2.95)	0.48
Previous or current IDU	1.87 (0.49 - 7.19)	0.36	2.07 (0.15 - 28.82)	0.59	3.14 (0.26 - 37.46)	0.37
AIDS	1.60 (0.44 - 5.79)	0.48	0.83 (0.06 - 11.37)	0.89	2.94 (0.23 - 37.30)	0.41
Baseline sqrt CD4	0.99 (0.89 - 1.09)	0.84	1.02 (0.84 - 1.23)	0.85	0.87 (0.66 - 1.14)	0.30
Baseline log HIV-1 RNA	0.79 (0.04 - 16.01)	0.88	0.92 (0.02- 53.13)	0.97	0.03 (0.00 - 36.86)	0.34
AZT or d4T backbone	1.79 (0.52- 6.13)	0.35	1.76 (0.19- 15.91)	0.61	9.75 (0.34- 276.9)	0.18
Adherence (per increase in stratum)	1.23 (0.57 - 2.65)	0.59	0.65 (0.11 - 3.73)	0.63	8.38 (1.26 - 55.70)	0.03

* Including only those patients on a regimen with a 3TC or FTC backbone (n=137)

OR=odds ratio, IDU=injecting drug use, PI/r=ritonavir-boosted protease inhibitors, NRTI= nucleoside reverse transcriptase inhibitor, AZT=zidovudine, d4T=stavudine OR: odds ratio; IDU: injecting drug use; AZT: zidovudine

In the NNRTI group, only the development of any IAS-USA mutations showed a statistically significant linear univariable association with adherence (1.61 [1.06-2.46]). The association between NNRTI mutations (1.46 [0.94-2.23]) and M184V (1.01 [0.49-2.08]) and adherence did not reach statistical significance. In multivariable models provided in Table 4, the emergence of any IAS-USA mutation remains significantly associated with adherence (1.66 [1.04-2.67]). In addition, the association between adherence and NNRTI mutations becomes significant after adjustment for confounders (1.65 [1.00-2.70]). In multivariable models for any IAS-USA mutation and NNRTI mutation, currently being on a regimen with ABC, TDF or didanosine (ddI) resulted in a decreased risk for the development of mutations. The majority of those treatments contained TDF (143 of 204, 70%), which is more potent, can be given once daily, is better tolerated than zidovudine (AZT) [35], and hence may have led to fewer problems with adherence and less drug resistance [36].

**Table 4:** Multivariable logistic regression models for the development of mutations in individuals on NNRTI regimens.

**Variable**			**Any IAS-USA mutation**		**Any NNRTI mutation**		**Any M184V/I mutation**	
			**Multivariable OR**	**p-value**	**Multivariable OR**	**p-value**	**Multivariable OR**	**p-value**
Female			1.63 (0.70 - 3.78)	0.26	1.17 (0.48 - 2.85)	0.72	2.22 (0.76 - 6.51)	0.15
Age - per 10 years			0.76 (0.50 - 1.15)	0.19	0.79 (0.50 - 1.23)	0.30	0.97 (0.56 - 1.71)	0.93
Previous or current IDU			0.46 (0.09 - 2.43)	0.36	0.56 (0.11 - 3.08)	0.52	1.85 (0.29 - 11.79)	0.52
AIDS			0.83 (0.36 - 1.95)	0.68	0.78 (0.32 - 1.94)	0.62	0.98 (0.31 - 3.13)	0.98
Baseline sqrt CD4			0.96 (0.91 - 1.03)	0.28	0.94 (0.87 - 1.01)	0.07	0.95 (0.87 - 1.04)	0.29
Baseline log HIV-1 RNA			0.71 (0.17 - 2.91)	0.63	0.86 (0.17 - 4.21)	0.85	0.84 (0.07 - 9.51)	0.89
NRTI backbone	3TC or FTC		0.36 (0.10 - 1.29)	0.12	0.26 (0.07 - 1.00)	0.05	1.53 (0.10 - 23.81)	0.76
	AZT or d4T		0.51 (0.16- 1.67)	0.27	0.39 (0.11- 1.36)	0.14	2.31 (0.37- 14.49)	0.37
	ABC TDF or ddI		0.19 (0.05 - 0.68)	0.01	0.13 (0.03 - 0.53)	0.004	0.23 (0.03 - 1.85)	0.17
Adherence (per increase in stratum)			1.66 (1.04 - 2.67)	0.03	1.65 (1.00 - 2.70)	0.05	1.47 (0.79 - 2.76)	0.23

OR=odds ratio, IDU=injecting drug use, NNRTI= non-nucleoside reverse transcriptase inhibitor , NRTI= nucleoside reverse transcriptase inhibitor, 3TC= lamivudine, FTC=emtricitabine, AZT=zidovudine, d4T=stavudine, ABC=abacavir, TDF=tenofovir, ddI=didanosine

### Associations of HIV RNA and adherence

We further investigated the impact of adherence on levels of HIV RNA measured at time of genotypic testing in our sample. In the PI/r group, average levels of HIV RNA log_10_ copies/mL (standard deviation) were increasing with lower adherence and reached 2.8 (1.5) log copies/mL in the 100% adherence group, 3.2 (1.5) log copies/mL in the 95-99% group and 3.7 (0.9) log copies/mL in the <95% group. Consequently, there was a statistically significant linear trend of 0.5 [95% confidence interval 0.1-0.8] log_10_ copies/mL per lower adherence stratum (R^2^=0.053; F-test p=0.004). No such relationship was observed in the NNRTI group, where HIV RNA levels remained approximately constant across all adherence strata (in descending order) with 3.6 (1.3), 3.6 (0.9), and 4.0 (0.9) log_10_ copies/mL. Neither of these adherence group means differed significantly (F-test p=0.33). The sensitivity analysis yielded almost identical results. For the PI/r group a decrease in adherence was associated with a 0.46 [95% confidence interval 0.1-0.8] log_10_ copies/mL increase in HIV RNA, whereas no association was observed for the NNRTI group.

### Durability of regimens

We compared the durability of the regimens and found the viral failure rate to be significantly higher in those on NNRTI compared to PI (78.0% vs. 47.2%, p<0.001). In those who experienced virological failure, the average durability of the regimen was 21 and 15 months in NNRTI and PI/r respectively (p=0.039) Within the NNRTI group, the average durability of those on regimens with EFV was slightly longer than those on NVP (22 versus 19 months) but the difference was not statistically significant. 

## Discussion

In this carefully selected data set of genotypic drug resistance tests and adherence measurements from individuals treated with potent combination antiretroviral therapies, we observed associations of NNRTI drug resistance emergence with levels of prior self- reported adherence, while associations for PI/r regimens varied according to type of resistance mutation. Therapies containing boosted PI seemed more forgiving to incomplete compliance compared with NNRTI-based therapies, which allowed much higher levels of resistance emergence, even at adherence levels of 95% and higher. In the NNRTI group, levels of resistance defined by the presence of at least one IAS-USA mutation increased markedly from the highest (29%) to the lowest adherence stratum (50%). Not surprisingly, many of those individuals from the NNRTI group with at least 1 new mutation also harboured NNRTI resistant viruses (ranging from 83% - 87%, Table 2), which reflects the fact that NNRTI resistance mutations tend to require few nucleotide changes, incur a small fitness cost on the virus, and have a large resistance impact when compared with PI mutations. For example, 48% to 64% of new NNRTI mutations consisted of the amino acid change K103N (data not shown), which is known to emerge rapidly and to confer full resistance to EFV and NVP. In contrast, there was little statistical evidence for increases in resistance levels at lower adherence strata when a boosted PI was used, although these analyses were somewhat limited in power due to the low frequency of resistance emergence on boosted PI regimens in general. 

Further noteworthy, we found associations of HIV RNA levels with adherence strata in the PI/r group, but not in the NNRTI group. Most likely, this observation is tied to the fact that a single NNRTI mutation can render NNRTI-drugs like efavirenz or nevirapine impotent, whereas several mutations are usually required to have a strong impact on PI susceptibility. Therefore, this similarity in HIV RNA levels across adherence strata in the NNRTI group possibly reflects the higher degree of resistance in NNRTI regimens and consequently the lower residual efficacy of those treatments. This notion is supported by the finding that the Stanford cumulative genotypic sensitivity scores of the drug combination at the time of genotyping were generally higher in the PI/r group than in the NNRTI group and that there was a trend for lower scores (i.e. more resistance) with lower adherence in the PI/r group but not in the NNRTI group.

Taken together, these observations suggest that good adherence may be beneficial even with virologically failing PI/r regimens, because this may prevent the accumulation of further resistance mutations and can therefore help to preserve future drug options. In contrast, adherence levels seem to have little impact on NNRTI treatments once the first mutations, and NNRTI mutations in particular, have emerged [37]. 

Our data are largely consistent with previous studies showing that antiretroviral classes may have different adherence–resistance relationships. Gallego et al [38] found resistance in PIs was limited to individuals reporting more than 90% adherence. Parienti et al. found that NNRTI resistance is associated with interruptions of therapy [22]. Sethi et al. [23] and Maggiolo et al [14] found resistance occurring in those on NNRTIs at lower levels of adherence than that observed in patients who develop resistance to PIs. Longitudinal studies by Bangsberg et al. and Miller et al. found that increasing adherence independently predicts the rate of accumulation of drug resistance mutations among patients with persistent detectable viraemia [10,39]. Collectively, these studies have shown that the greatest risk for resistance is in patients with high levels of adherence and incomplete viral suppression, and this relationship is strongest for PI-based therapy. Our analyses complement previous work by additionally considering the impact of adherence on HIV RNA levels and genotypic sensitivity scores upon virological failure. The importance of viral replication on HIV-related, but not AIDS-defining morbidities has been demonstrated previously [40], and our data suggest that improved adherence on failing PI/r regimens may still contribute to partial viral control, but not with failing NNRTI treatments.

Several limitations should be noted about this analysis. Despite drawing from large observational databases including almost 6800 genotypic drug resistance tests from drug exposed individuals, the final numbers of patients included in this analysis were small, but of the same order of magnitude of other studies [6,14,15]. Owing to the observational nature of this study, the treatments received were not randomized and the collection of genotypic data was not strictly enforced by study protocols. Moreover, we did not strictly analyze GRTs from first-line failures, but included additional data to increase sample size (Figure 1). We can therefore not exclude residual confounding in our analyses Furthermore, our adherence assessments are based on self-report, which is prone to recollection biases or over-reporting. Although we ask about consecutive missed doses, self-report data may not be sensitive to important adherence patterns, such as treatment interruptions. Moreover, not all individuals had genotypic resistance data collected prior to treatment initiation or at time of the first virological failure available, and some mutations could potentially have been transmitted. However, extensive longitudinal assessments of transmitted drug resistance in Switzerland have shown that the presence of drug resistance mutations before therapy initiation is still relatively rare [41]. 

Our observations have important clinical implications. It is self-evident that good adherence promotes viral suppression and reduces the emergence of resistance mutations. In this analysis, we also observed evidence that adherence levels influenced resistance levels of virologically failing or failed PI/r regimens. Thus, even when partial resistance has occurred, good adherence levels may slow down or even stop progression to higher resistance levels, thereby possibly extending the durability of PI/r regimens [37]. This effect may have great relevance in settings of limited antiretroviral drug availability and virological monitoring. In contrast, current WHO-recommended first-line treatments are based on NNRTI for cost reasons, which must be considered sub-optimal from the standpoint of emergence of resistance. In NNRTI therapies, resistance emergence is more likely with incomplete drug intake and cannot be limited by improved adherence later on. Thus, even temporary slips in adherence carry a substantial risk for losing the full drug combination to resistance.

In summary, our data suggest that promoting adherence may still be worthwhile for individuals receiving virologically failing PI/r regimens for damage control, but failing NNRTI regimens should be switched immediately.

## Supporting Information

Table S1 **Development of new mutation by adherence level and drug class for individuals who either started their first antiretroviral therapy ever or who started a new class of ART and who had a prior on-treatment genotypic test result available (PI/r: n= 124; NNRTI: n= 111).**
(DOCX)
