# Supplementary material for: Adherence as a Predictor of the Development of Class-Specific Resistance Mutations: The Swiss HIV Cohort Study
Source: PLoS One. 2013 Oct 16;8(10):e77691. doi: 10.1371/journal.pone.0077691 (PMC3797701; doi:10.1371/journal.pone.0077691)
Supplement: Table S1 — Development of new mutation by adherence level and drug class for individuals who either started their first antiretroviral therapy ever or who started a new class of ART and who had a prior on-treatment genotypic test result available (PI/r: n= 124; NNRTI: n= 111). (DOCX) [file pone.0077691.s001.docx]

**Supplementary Information**:

**Table S1**: Development of new mutation by adherence level and drug class for individuals who either started their first antiretroviral therapy ever or who started a new class of ART and who had a prior on-treatment genotypic test result available (PI/r: n= 124; NNRTI: n= 111)

| **Adherence Level** | **PI/r** | **NNRTI** |
| --- | --- | --- |
| **Any IAS-USA mutation** | | |
| 100% | 5/93 (5.4) | 14/77 (18.2) |
| 95-99% | 0/16 (0) | 5/18 (27.8) |
| <95% | 1/15 (6.7) | 3/16 (18.9) |
| **Any mutation against group-specific drug (PI/r or NNRTI)** | | |
| 100% | 0/93 (0) | 11/77 (14.3) |
| 95-99% | 0/16 (0) | 3/18 (16.7) |
| <95% | 0/15 (0) | 2/16 (12.5) |
| **New emergence of M184V/I** | | |
| 100% | 1/52 (1.9) | 5/49 (10.2) |
| 95-99% | 0/9 (0) | 1/14 (7.1) |
| <95% | 1/9 (11.1) | 0/9 (0) |

NNRTI= non-nucleoside reverse transcriptase inhibitor , PI/r=ritonavir-boosted protease inhibitors
